# Phospholipids and insulin resistance in psychosis: a lipidomics study of twin pairs discordant for schizophrenia

**DOI:** 10.1186/gm300

**Published:** 2012-01-18

**Authors:** Matej Orešič, Tuulikki Seppänen-Laakso, Daqiang Sun, Jing Tang, Sebastian Therman, Rachael Viehman, Ulla Mustonen, Theo G van Erp, Tuulia Hyötyläinen, Paul Thompson, Arthur W Toga, Matti O Huttunen, Jaana Suvisaari, Jaakko Kaprio, Jouko Lönnqvist, Tyrone D Cannon

**Affiliations:** 1 VTT Technical Research Centre of Finland, Tietotie 2, Espoo, FI-02044 VTT, Finland; 2 Department of Psychology, University of California Los Angeles, Los Angeles, 5586 Franz Hall, Los Angeles, CA 90089, USA; 3 Department of Mental Health and Alcohol Research, National Institute for Health and Welfare, Lintulahdenkuja 4, Helsinki, FI-00271, Finland; 4 Department of Psychiatry and Human Behavior, University of California Irvine, 5251 California Avenue, Irvine, CA 92697, USA; 5 Department of Neurology and Laboratory of NeuroImaging, University of California Los Angeles, 635 Charles E. Young Drive South, Los Angeles, CA 90095-7332, USA; 6 Department of Public Health, University of Helsinki, Haartmaninkatu 8, Helsinki, FI-00290, Finland; 7 Department of Psychiatry, Helsinki University Hospital, Välskärinkatu 12, Helsinki, FI-00029, Finland; 8 Department of Psychiatry and Biobehavioral Sciences, University of California Los Angeles, 760 Westwood Plaza, Los Angeles, CA 90095, USA

## Abstract

**Background:**

Several theories have been proposed to conceptualize the pathological processes inherent to schizophrenia. The 'prostaglandin deficiency' hypothesis postulates that defective enzyme systems converting essential fatty acids to prostaglandins lead to diminished levels of prostaglandins, which in turn affect synaptic transmission.

**Methods:**

Here we sought to determine the lipidomic profiles associated with schizophrenia in twin pairs discordant for schizophrenia as well as unaffected twin pairs. The study included serum samples from 19 twin pairs discordant for schizophrenia (mean age 51 ± 10 years; 7 monozygotic pairs; 13 female pairs) and 34 age and gender matched healthy twins as controls. Neurocognitive assessment data and gray matter density measurements taken from high-resolution magnetic resonance images were also obtained. A lipidomics platform using ultra performance liquid chromatography coupled to time-of-flight mass spectrometry was applied for the analysis of serum samples.

**Results:**

In comparison to their healthy co-twins, the patients had elevated triglycerides and were more insulin resistant. They had diminished lysophosphatidylcholine levels, which associated with decreased cognitive speed.

**Conclusions:**

Our findings may be of pathophysiological relevance since lysophosphatidylcholines, byproducts of phospholipase A2-catalyzed phospholipid hydrolysis, are preferred carriers of polyunsaturated fatty acids across the blood-brain barrier. Furthermore, diminishment of lysophosphatidylcholines suggests that subjects at risk of schizophrenia may be more susceptible to infections. Their association with cognitive speed supports the view that altered neurotransmission in schizophrenia may be in part mediated by reactive lipids such as prostaglandins.

## Background

Several theories have been proposed to conceptualize the pathological processes inherent to schizophrenia, among others altered neurotransmission, autoimmune dysfunction and dysregulation of inflammation [1,2]. The phospholipid hypothesis suggests that deficient uptake or excessive breakdown of membrane phospholipids or changes in membrane phospholipid composition may be associated with schizophrenia [3]. The hypothesis is supported by studies finding lipid abnormalities both in postmortem gray and white matter samples and from peripheral red blood cells of patients with schizophrenia [4]. Moreover, phospholipase A2 activity is increased particularly in the first-onset patients with schizophrenia and associates with structural brain changes, particularly in the prefrontal cortex and thalamus [5]. Nevertheless, the evidence for the phospholipid hypothesis is not conclusive [6]. Prior to the phospholipid hypothesis, a prostaglandin deficiency hypothesis was proposed [7]. Phospholipid abnormalities and prostaglandin deficiency may be related since prostaglandins are synthesized via hydrolysis of phosphatidylcholine (PC) by action of phospholipase A2, with another byproduct of this process being lysophosphatidylcholines (lysoPCs).

Directly addressing the phospholipid and prostaglandin hypotheses by measuring the relevant phospholipids and prostaglandins has been difficult until recently. Even today, reliable measurements of prostaglandins from clinical samples are-despite the availability of sensitive instruments and analytical techniques-very challenging to make due to sensitivity of prostaglandins to sample collection and pre-treatment. However, the emergence of metabolomics, and with it also lipidomics [8], a global study of lipids, has enabled comprehensive and sensitive measurement of molecular lipids, including phospholipids in biofluids and tissues. Here we sought to determine the lipidomic profiles associated with schizophrenia in twin pairs discordant for schizophrenia as well as unaffected twin pairs. The use of twin pairs discordant for the disorder enables separation of lipidomic profiles associated with genetic liability to disorder from those associated with illness expression or treatment.

## Materials and methods

### Participants and clinical evaluation

Same-sex twin pairs discordant for schizophrenia were identified from Finnish national registers, as described in detail in Cannon *et al*. [9], and interviewed with the Structured Clinical Interview for the Diagnostic and Statistical Manual of Mental Disorders (DSM)-III-R [10]; diagnoses were assigned according to the DSM-IV [11]. The procedures for determining diagnoses and zygosity have been presented in detail by Cannon *et al*. [12]. Only patients with a confirmed diagnosis of schizophrenia who were not inpatients at the time were asked to participate in the blood sampling. Healthy control twin pairs selected from the Finnish Twin Cohort were matched by age and gender. Demographic information is presented in Table 1.

**Table 1:** Demographic and metabolic characteristics of the study population, shown for patients, their unaffected co-twins, patients and their co-twins combined (discordant twin pairs), and the controls

Characteristic	Controls	Unaffected co-twins	Patients	*P *(F-test)
*N*	34	19	19	
Monozygotic	20 (10 pairs)	7	7	
Female	24	13	13	
Age (years)^a^	53.4 (50.2, 56.6)	51.0 (46.4, 55.6)	51.0 (46.4, 55.6)	
Diabetes	0	1	7	
Antipsychotic medication (atypical antipsychotics)^b^	0	0	15 (14)	
Cholesterol medication	2	0	6	
BMI	25.4 (23.8, 27.0)**	25.7 (24.0, 27.5)**	29.2 (26.5, 31.9)	0.020
Insulin^c^	7.7 (5.9, 9.9)	8.9 (6.9, 11.6)*	14.1 (9.1, 21.7)	0.085
Glucose^c^	5.07 (4.78, 5.38)	5.30 (4.54, 6.19)	5.76 (4.87, 6.80)	0.40
HOMA-IR^c^	1.72 (1.30, 2.29)	2.11 (1.52, 2.93)**	3.61 (2.22, 5.86)	0.062

^a^Data for this and other variables are shown as mean (95% confidence interval). ^b^According to self-report. ^c^Geometric means and confidence intervals; statistics estimated from log10-transformed data. *P < 0.1, **P < 0.05 (post-hoc analysis using Tukey all-pair comparisons versus the patient group; no comparisons were significant between the controls and unaffected co-twins). BMI, body mass index; HOMA-IR, Homeostasis Model Assessment index.

Symptom severity was quantified on the Scale for the Assessment of Negative Symptoms (SANS) and the Scale for the Assessment of Positive Symptoms (SAPS) [13,14]. Patients had, on average, mild to moderate negative symptoms (SANS mean ± standard deviation 1.3 ± 0.9, with personal maximums of 2.5 ± 1.0) and moderate positive symptoms (SAPS mean ± standard deviation 2.0 ± 0.8, with personal maximums of 3.5 ± 0.9). Two patients met criteria for remission [15].

All participants took part in an extensive neurocognitive assessment with established tasks known to be sensitive to cognitive deficits in schizophrenia. The cognitive tasks used and the rationale for their inclusion have been presented before in detail [16]. Cognitive results are presented in Additional file 1.

The study protocol was reviewed and approved by the institutional review boards of the National Public Health Institute, Helsinki, Finland, and the University of California-Los Angeles, and all participants signed institutional review board-approved informed consent forms.

### Image acquisition and analysis

T1-weighted MPRAGE MRI volumes were acquired on a 1.5-Tesla scanner (Siemens, Iselin, NJ, USA) in the Department of Radiology at Helsinki University Central Hospital (128 contiguous 1.2-mm slices in the sagittal plane, repetition time 11.4, echo time 4.4, 256 × 256 matrix). A radio-frequency bias field-correction algorithm eliminated intensity drifts caused by scanner field inhomogeneity. Cerebral images were automatically extracted from the scans, manually edited, and classified into gray matter, white matter, and cerebrospinal fluid components.

High-resolution surface models of cerebral hemispheres were generated automatically for each participant. Raters blind to diagnostic and demographic information drew 36 gyral and sulcal landmarks, representing the primary gyral pattern, as three-dimensional curves on each of the surface models by using a detailed anatomical protocol [17,18]. Raters were trained on a set of six brains until they were able to trace landmarks with three-dimensional deviations no greater than 4 mm everywhere and 2 mm on average when compared with 'gold standard' tracing.

The hemispheric surfaces were elastically warped to each other based on matching individual curves to their corresponding average curves, and three-dimensional deformation fields were obtained. A local measurement termed gray-matter density (GMD) was calculated for each individual, whereby the proportion of gray matter is measured in a sphere of fixed radius (15 mm) around each cortical point. This proportion thus reflects the amount of local gray matter in the sphere. Maps representing the variability in GMD across cortex were then generated for all individuals and were used in further analysis.

The GMD data were available from 34 participants (9 patients, 9 co-twins, and 16 controls). The imaging and neurocognitive data were collected on average 5 years prior to blood sampling for this specific study.

### Insulin and glucose assays

Serum concentrations of insulin and glucose were analyzed at the Finnish National Institute for Health and Welfare using Architect ci8200 analyzer (Abbott Laboratories, Abbott Park, IL, USA). The inter-assay coefficient of variation of insulin varied from 1.5% (low level control, 26 mU/L) to 2.7% (high level control, 166 mU/L). The inter-assay coefficient of variation of glucose was 2.0%.

### Lipidomic analysis

EDTA-blood samples (10 ml) were centrifuged at 3,200 rpm (1,600G) for 15 minutes at room temperature within 2 hours of blood sampling. Serum was separated and stored at -80°C. For lipidomics profiling, 10 μl aliquots of serum were used. The samples were mixed with 10 μl of 0.9% sodium chloride in Eppendorf tubes, spiked with a standard mixture consisting of 10 lipids (0.2 μg/sample; lysophosphatidylcholine LPC(17:0/0:0), phosphatidylcholine PC(17:0/17:0), phosphatidylethanolamine PE(17:0/17:0), phosphatidylglycerol PG(17:0/17:0), ceramide Cer(d18:1/17:0), phosphatidylserine PS(17:0/17:0), phosphatidic acid PA(17:0/17:0), monoglyceride MG(17:0/0:0/0:0), diglyceride DG(17:0/17:0/0:0), triglyceride TG(17:0/17:0/17:0)) and extracted with 100 μl of chloroform/methanol (2:1). After vortexing (2 minutes) and standing (1 hour) the tubes were centrifuged at 10,000 rpm for 3 minutes and 60 μl of the lower organic phase was separated and spiked with a standard mixture containing three labeled lipids (0.1 μg/sample; LPC(16:1/0:0-D_3_), PC(16:1/16:1-D_6_), TG(16:0/16:0/16:0-^13^C3)).

Lipid extracts were analyzed in a randomized order on a Waters Q-Tof Premier mass spectrometer combined with an Acquity Ultra Performance LC™ (UPLC). The column (at 50°C) was an Acquity UPLC™BEH C18 1 × 50 mm with 1.7 μm particles. The solvent system included 1) ultrapure water (1% 1 M NH_4_Ac, 0.1% HCOOH) and 2) liquid chromatography/mass spectrometry grade acetonitrile/isopropanol (5:2, 1% 1 M NH_4_Ac, 0.1% HCOOH). The gradient started from 65% A/35% B, reached 100% B in 6 minutes and remained there for the next 7 minutes. There was a 5-minute re-equilibration step before the next run. The flow rate was 0.200 ml/minute and the injected amount was 1.0 μl (Acquity Sample Organizer). Reserpine was used as the lock spray reference compound. The lipid profiling was carried out using ESI+ mode and the data were collected at mass range of m/z 300 to 1,200 with a scan duration of 0.2 s. The data were processed by using MZmine 2 software [19] and the lipid identification was based on an internal spectral library.

The data were normalized using one or more internal standard representatives of each class of lipid present in the samples [20]: the intensity of each identified lipid is normalized by dividing it with the intensity of its corresponding standard and multiplying it by the concentration of the standard. All monoacyl lipids except cholesterol esters, such as monoacylglycerols and monoacylglycerophospholipids, were normalized with PC(17:0/0:0), all diacyl lipids except ethanolamine phospholipids were normalized with PC(17:0/17:0), all ceramides with Cer(d18:1/17:0), all diacyl ethanolamine phospholipids with PE(17:0/17:0), and TG and cholesterol esters with TG(17:0/17:0/17:0). Other (unidentified) molecular species were calibrated with PC(17:0/0:0) for retention time < 300 s, PC(17:0/17:0) for retention time between 300 s and 410 s, and TG(17:0/17:0/17:0) for higher retention times.

### Statistical analysis of lipidomics data

Linear mixed models were applied to the twin-pair data using the R statistical language v2.13 [21], implemented in R package nlme. Intra-pair correlations were treated as random effects in the model. The overall group difference was tested using F-statistic and the post-hoc analyses were done using Tukey's all-pair comparisons. The adjusted *P*-value for each comparison was computed using multiple testing procedures under free combinations (R package multcomp, option 'free' in the summary function).

Individual metabolite levels were visualized using the beanplot [22] algorithm implemented in R. A beanplot provides information on the mean metabolite level within each group and the density of the data-point distribution and also shows individual data points.

The data were rescaled into zero mean and unit variance to obtain metabolite profiles comparable to each other for clustering. Bayesian model-based clustering was applied on the scaled data to group lipids into clusters. The analyses were performed using the MCLUST [23] method, implemented in R [21].

### Statistical analysis of image data

To reduce GMD data and facilitate network analysis, independent component analysis was applied to GMD maps from all individuals, using the FastICA algorithm in the Modular toolkit for Data Processing [24]. Each independent component was examined on three-dimensional cortical surface maps and the weights for all individuals in the mixing matrix were used in the network analysis.

### Partial correlation network analysis

Construction of the dependency network for selected variables was performed using undirected Gaussian graphical Markov networks that represent *q*-order partial correlations between variables (the so called QPGRAPH method), implemented in an R package 'qpgraph' from the Bioconductor project [25]. In these networks non-missing edges denote non-zero partial correlations between pairs of variables and thus imply direct interactions. The network was visualized using yED graphical editor [26].

## Results

### Associations of the global lipidome with schizophrenia

Using the lipidomics platform, a total of 530 molecular lipids were detected, of which 250 were identified (the full dataset is provided in Additional file 2). Due to a high degree of co-regulation among the lipids, one cannot assume that all the 530 measured lipids are independent. The global lipidome was therefore first surveyed by clustering the data into a subset of clusters using Bayesian model-based clustering. Lipidomic platform data were decomposed into seven clusters. Descriptions of each cluster and representative lipids are given in Table 2. As expected, the division of clusters to a large extent follows different lipid functional or structural groups.

**Table 2:** Description of lipid clusters obtained from lipidomics platform

Cluster name	Cluster size	Description	Representative abundant lipids	*P *(F-test)
LC1	68	Mainly ether lipids	PE(36:6e), PC(34:4e)	0.39
LC2	211	Sphingomyelins, major phospholipids	SM(d18:1/18:0), SM(d18:1/24:1), PC(34:2)	0.69
LC3	49	PUFA-containing PCs and PEs	PC(38:6), PE(38:5)	0.91
LC4	25	LysoPCs	lysoPC(18:0), lysoPC(20:3)	0.096^a^
LC5	76	Abundant TGs	TG(16:0/18:1/18:1), TG(18:1/18:2/18:1)	0.061^b^
LC6	35	Long-chain TGs; PUFA-containing TGs	TG(56:6), TG(58:8), TG(58:9)	0.25
LC7	66	Mainly shorter-chain SFAs and MUFA-containing TGs	TG(14:0/16:0/18:1), TG(14:0/16:0/18:0)	0.65

^a^Tukey's all-pair comparison: *P *= 0.12 (patients versus co-twins), *P *= 0.078 (co-twins versus controls), *P *= 0.56 (patients versus controls). ^b^Tukey's all-pair comparison: *P *= 0.039 (patients versus co-twins), *P *= 0.44 (co-twins versus controls), *P *= 0.24 (patients versus controls). LC, LC, lipid cluster; lysoPC, lysophosphatidylcholine; MUFA, monounsaturated fatty acid; PC, phosphatidylcholine; PE, phosphatidylethanolamine; PUFA, polyunsaturated fatty acid; SFA, saturated fatty acid; SM, sphingomyelin; TG, triglyceride.

**Figure 1:**
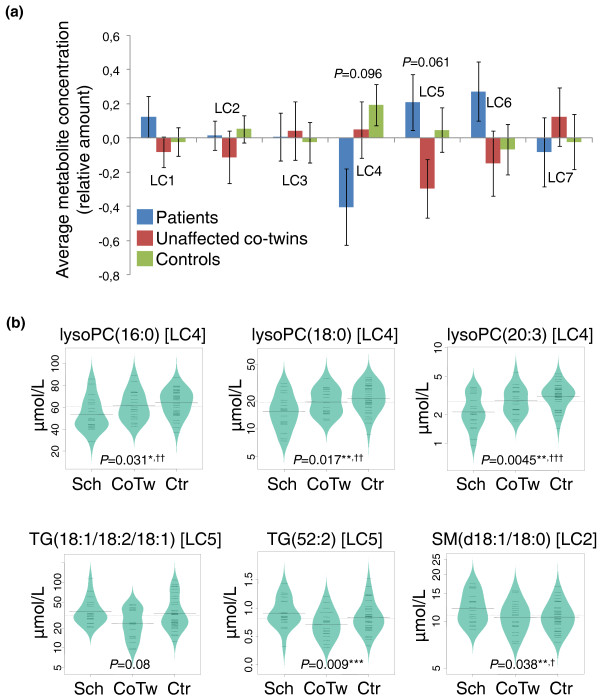
**Lipid levels across the three study groups, as obtained from the lipidomics platform**. **(a) **Mean lipid levels within each cluster. Error marks show standard error of the mean. The F-test (across the three groups) *P*-values are shown. **(b) **Profiles of selected representative abundant lipids from three clusters. lysoPC(16:0) and lysoPC(18:0) are the two most abundant lysophosphatidylcholines measured in plasma. The lipid levels are shown as beanplots [22], which provide information on the mean level (solid line), individual data points (short lines), and the density of the distribution. Note that the concentration scale in beanplots is logarithmic. Tukey all-pair comparison post-hoc test: patients versus co-twins (*P < 0.1, **P < 0.05, ***P < 0.001), patients versus controls (^†^*P *< 0.1, ^††^*P *< 0.05, ^†††^*P *< 0.01). No significant differences were found when comparing co-twins and controls. CoTw, unaffected co-twins; Ctr, controls; Sch, patients.

As shown in Figure 1a, the major differences observed were in clusters LC4 and LC5, corresponding to lysoPCs and abundant TGs, respectively. LysoPCs were diminished in schizophrenia patients compared to their co-twins as well as healthy controls (Figure 1b). TGs from LC5 were elevated in patients compared to their co-twins (Figure 1b), but there were no differences between the healthy twins from schizophrenia-discordant pairs and controls. In the large phospholipid cluster LC2, while no changes were observed for the cluster overall, the major lipid-class-specific changes were observed for sphingomyelins, which were elevated in patients compared to their healthy co-twins (Figure 1b shows one representative abundant sphingomyelin).

The observed differences in lipidomes were accompanied by differences in metabolic and body composition characteristics (Table 1). Patients had higher body mass index (BMI), insulin (at marginal significance level) and Homeostasis Model Assessment index (HOMA-IR) than their unaffected co-twins. No differences in glucose levels were observed.

### Dependency analysis

Next, we investigated the associations between the observed metabolic and body compositional changes and the earlier acquired neurocognitive and image data. The included 21 neurocognitive variables are listed in Additional file 1. MRI data were first decomposed by independent component analysis into 34 independent components (ICs), of which 14 were included in the dependency network analysis. The selection criteria were (1) known relevance of the represented region to schizophrenia or (2) differences between the groups were at the *P *< 0.15 level. The selected ICs are described in Additional file 3. In addition to the seven lipid clusters (LCs; Figure 1), other variables included in the analysis were age, BMI, fasting plasma glucose and insulin as well as HOMA-IR.

To distinguish direct and indirect interactions of these 49 variables, we utilized the QPGRAPH method, which has been previously applied to study gene regulatory networks based on microarray data [25] as well as in our earlier population-based metabolomics study of psychoses [27]. QPGRAPH uses partial correlations as a measure of dependency and builds an undirected Gaussian graphical model where the variables are connected if and only if their partial correlation is significantly non-zero. Unlike the commonly applied pairwise measure of associations such as Pearson correlation coefficients, partial correlation provides a stronger criterion for dependency by adjusting for the confounding effects and thus removes spurious associations to a large extent.

**Figure 2:**
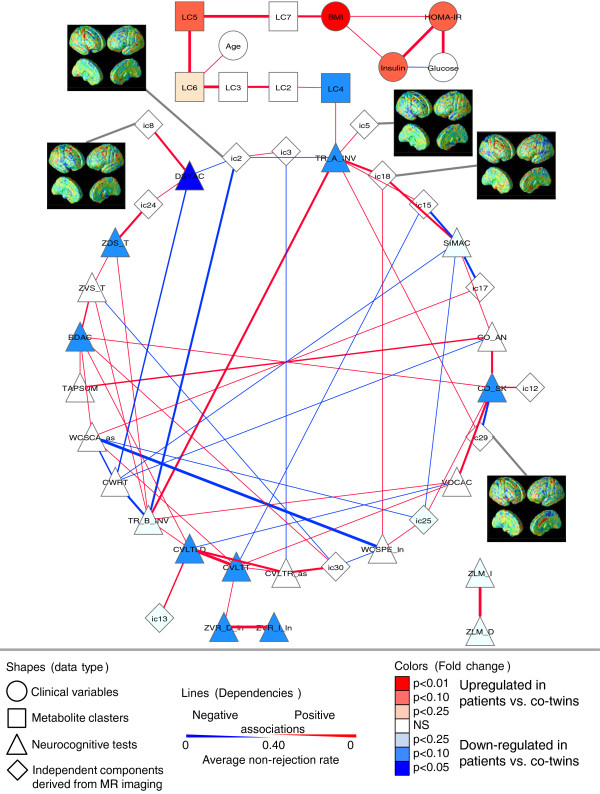
**Dependency network of variables related to schizophrenia**. The network was constructed from the selected clinical, lipid cluster, MR image (with selected independent components shown), and neurocognitive assessment data. Node shapes represent different types of variables, node color corresponds to significance and direction of regulation comparing patients versus unaffected co-twins, and line width is proportional to strength of dependency. The cutoff for the presence of edge was set at *β *= 0.40 by the average non-rejection rate, that is, an edge in the graph was tested positive in 40% of the 500 samplings. The existing edges should be interpreted as direct associations between the pairs of variables. HOMA-IR, Homeostasis Model Assessment index; NS, not significant.

**Figure 3:**
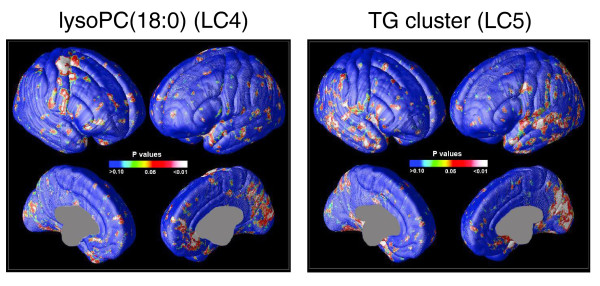
**Significant correlations between lipid levels and cortical gray matter density**. LysoPC(18:0) level is positively correlated with gray matter density mainly in right precentral gyrus, anterior cingulate areas, and medial parietal and occipital surfaces. The triglyceride cluster LC5 is positively correlated with gray matter density in lateral temporal surfaces on both sides and medial occipital and parietal surfaces on the right side. These results were confirmed using permutation tests.

As expected, the TG clusters LC5 to LC7 were strongly associated with the metabolic variables, including BMI and HOMA-IR (Figure 2). Network analysis did not reveal direct associations of TG clusters with any of the MRI-derived independent components, but the direct cortical mapping of the TG cluster LC5 revealed that this cluster is positively correlated with gray matter density in lateral temporal surfaces on both sides and medial occipital and parietal surfaces on the right side (Figure 3).

The lysoPC cluster LC4 was associated with neurocognitive variables related to cognitive speed, especially with Trail Making Task A (TMT-A) response time, and was also close to Trail Making Task B (TMT-B) response time, WAIS-R Digit Symbol score, and Stroop Color Word Response Time. TMT-A was associated with five GMD independent components, among which IC2 (right precentral areas) was increased in twin pairs discordant for schizophrenia compared to controls, and IC5 (superior temporal gyri) was decreased at a marginal significance level (Figure 2). Direct cortical mapping of abundant lysoPC(18:0) revealed positive correlation with gray matter density mainly in right precentral gyrus, anterior cingulate areas, and medial parietal and occipital surfaces (Figure 3).

## Discussion

Our findings based on a well-phenotyped cohort of twin pairs discordant for schizophrenia and healthy controls independently associate specific lipid abnormalities with schizophrenia and with genetic liability to schizophrenia as well as with specific intermediate phenotypes obtained from brain imaging and neurocognitive testing.

The observed elevation of triglycerides in schizophrenia patients in comparison to their healthy co-twins is in agreement with earlier findings that schizophrenia is associated with elevated fasting total triglycerides and insulin resistance [27,28]. This metabolic abnormality has been usually attributed to antipsychotic drug-specific side effects. However, there is growing evidence that elevated circulating insulin and insulin-related peptides and abnormal insulin response to glucose can be identified already in drug-naïve first-episode patients [29-32], suggesting that insulin signaling is impaired in schizophrenia [30,31].

In a recent lipidomic study of different lipoprotein fractions in people with varying degrees of insulin resistance we found that the lipids observed in LC5 are abundant in liver-produced very low density lipoprotein particles and are associated with insulin resistance [33]. In agreement with this, schizophrenia patients in the present study were insulin resistant. Additionally, the twins from pairs discordant for schizophrenia had a higher HOMA-IR index compared to healthy controls (Table 1), implying that these differences may not be due to antipsychotic medication use.

One should bear in mind that atypical antipsychotics used by a majority of patients in our study (Table 1) are known to have a strong effect on serum lipidomic profiles [34]. Consequently, with the data from the current study alone we are not able to control for the effect of antipsychotic medication used on lipidomic profiles, specifically on the elevated triglycerides in the patients. However, we have already shown in our earlier study that triglycerides are significantly elevated in schizophrenia patients even after correcting for antipsychotic medication use and metabolic co-morbidity [27]. Furthermore, previous studies finding elevated insulin and related peptides have been done on antipsychotic-naïve first-episode patients [29-31]. Together, our data indicate that schizophrenia is characterized by insulin resistance and consequently enhanced hepatic very low density lipoprotein production [35] and elevated serum concentrations of specific triglycerides.

LysoPCs were diminished in schizophrenia patients compared to their co-twins and to healthy controls. These changes were associated with decreased cognitive speed. The observed changes are particularly remarkable because elevated lysoPCs commonly positively associate with obesity and insulin resistance, as we have shown in a study of monozygous twins pairs discordant for weight [36]. Despite having measured lysoPC from serum, its observed variation may have direct implications for the lipid metabolism in the brain since lysoPC is a preferred carrier of polyunsaturated fatty acids across the blood-brain barrier [37]. Polyunsaturated fatty acids are important for the development of normal brain function [3]. In individuals at ultra-high risk of a psychotic disorder, dietary intake of long chain ω-3 fatty acids also reduces the risk of progression to the disease [38].

One potential clue about the pathogenic relevance of our findings comes from the negative association of rheumatoid arthritis with schizophrenia. Oken and Schulzer [39] performed a meta-analysis of 16 studies encompassing over 70,000 patients with schizophrenia and over 350,000 patients with other major psychiatric conditions and concluded that rheumatoid arthritis 'occurs among schizophrenia patients at a rate of only 29 percent of the corresponding prevalence in other psychiatric patients' and is even lower than 29% when compared to the general population. Such negative association with schizophrenia led to the 'prostaglandin deficiency' hypothesis, which postulated that defective enzyme systems converting essential fatty acids to prostaglandins lead to diminished levels of prostaglandins [7], which as a consequence may lead to altered monoaminergic neurotransmission. Prostaglandins are-along with lysoPCs-byproducts of PC hydrolysis by phospholipase A2. In contrast to schizophrenia, lysoPC is increased in rheumatoid arthritis [40], thus supporting the view that prostaglandins and perhaps also lysoPC as a byproduct may play a protective role in schizophrenia. As an alternative hypothesis, based also on the evidence that infections in early life may play a role in etiology of schizophrenia [41], Torrey and Yolken [42] suggested that rheumatoid arthritis and schizophrenia 'share a common infectious and/or immune etiology and that once a person gets one of the diseases then they are relatively immune to the other'. In fact, these two hypotheses may be related. Prolonged low levels of lysoPC in early life increases susceptibility to infection [43]. Recent research in sepsis has also shown that lysoPC inversely correlates with the severity of infection [44] and that lysoPC administration to mouse models of sepsis protects them against lethality [45].

LysoPC is a major component of oxidized low density lipoprotein [46], but varying serum lysoPC concentrations may also be due to changes in high density lipoprotein (HDL) metabolism. LysoPC is particularly abundant in persons with high HDL cholesterol [47] and diminished levels of HDL cholesterol have been observed in patients with schizophrenia in a general population cohort [48]. Together, due to many sources influencing lysoPC concentration in blood, which may depend on the individual's genotype and lifestyle, it may be challenging to detect disease-specific changes of lysoPCs in the general population, such as in our recent study in a general population cohort [27]. The twin study design such as ours is a more suitable setting where genetic and environmental factors are better separated, and specific factors directly associated with the disease pathogenesis may be more sensitively detected.

As a potential limitation of our study, the imaging and neurocognitive data were collected on average 5 years prior to blood sampling for this specific study. However, there is recent evidence that correlation between age and cortical thickness is similar in patients and controls [49]. In a two-year longitudinal study of first-episode psychoses, neurocognitive deficits were found in place by onset of psychosis and remained stable over the course of the study [50]. We therefore consider that brain structure and cognitive performance would not change within this span to a degree or in a direction that would affect the conclusions being drawn from the data. However, the fact that the imaging data were available for only 34 out of 73 participants weakened the power of the association analysis of image data with other variables. Another potential limitation is the cross-sectional design of our study. Due to many sources affecting lysoPC concentration, longitudinal research in prodromal and early psychosis is needed to further elucidate its role in psychotic disorders. Finally, a more decisive separation of genetic versus environmental effects would have come from examining the monozygous twin pairs separately, but the numbers are too small to permit this.

## Conclusions

Our study suggests that insulin resistance and the related elevation of specific triglycerides are inherent features of schizophrenia. Furthermore, diminishment of lysoPCs suggests that subjects at risk of schizophrenia may be more susceptible to infections. Their association with cognitive speed supports the view that altered neurotransmission in schizophrenia is in part mediated by reactive lipids such as prostaglandins. Further studies are needed to confirm these findings as well as to establish the cause and mechanism of the altered lysoPC levels and their relation to psychosis.

## Abbreviations

BMI: body mass index; Cer: ceramide; DSM: Diagnostic and Statistical Manual of Mental Disorders; GMD: gray-matter density; HDL: high density lipoprotein; HOMA-IR: Homeostasis Model Assessment index; IC: independent component; LC: lipid cluster; lysoPC: lysophosphatidylcholine; MRI: magnetic resonance imaging; PC: phosphatidylcholine; PE: phosphatidylethanolamine; SANS: Scale for the Assessment of Negative Symptoms; SAPS: Scale for the Assessment of Positive Symptoms; TG: triglyceride.

## Competing interests

TDC reports that in the past 12 months he has served as a consultant to Rules-Based Medicine on serum-based biomarkers of schizophrenia. No other authors report biomedical financial interests or potential conflicts of interest.

## Authors' contributions

DS, JT and RV performed the statistical analysis. MO performed the statistical analysis, conceived of the study, participated in its design and coordination and drafted the manuscript. ST performed the statistical analysis and researched the primary clinical data. TSL and TH carried out lipidomic analyses. TGMvanE, PT, AWT and MOH participated in the study design. UM and JS researched the primary clinical data. JK, JL and TDC conceived of the study, participated in its design and coordination and drafted the manuscript. All authors read and approved the final manuscript for publication.

## Supplementary Material

Additional file 1 **Cognitive test scores**. Table showing cognitive test scores with group comparisons. Click here for file

Additional file 2 **Clinical and lipidomics data**. Dataset including clinical and lipidomics data. Click here for file

Additional file 3 **Selected independent components from MRI**. Table showing selected independent components derived from MRI, shown for patients, their co-twins, patients and their co-twins combined (discordant twin pairs), and the controls. Click here for file

## References

[B1] Keshavan MS Tandon R Boutros NN Nasrallah HA Schizophrenia, "just the facts": What we know in 2008: Part 3: Neurobiology. Schizoph Res 2008 106 89 107 10.1016/j.schres.2008.07.020 18799287

[B2] Potvin S Stip E Sepehry AA Gendron A Bah R Kouassi E Inflammatory cytokine alterations in schizophrenia: a systematic quantitative review. Biol Psychiatry 2008 63 801 808 10.1016/j.biopsych.2007.09.024 18005941

[B3] Horrobin DF The membrane phospholipid hypothesis as a biochemical basis for the neurodevelopmental concept of schizophrenia. Schizophr Res 1998 30 193 208 10.1016/S0920-9964(97)00151-5 9589514

[B4] Schwarz E Prabakaran S Whitfield P Major H Leweke FM Koethe D McKenna P Bahn S High throughput lipidomic profiling of schizophrenia and bipolar disorder brain tissue reveals alterations of free fatty acids, phosphatidylcholines, and ceramides. J Proteome Res 2008 7 4266 4277 10.1021/pr800188y 18778095

[B5] Smesny S Milleit B Nenadic I Preul C Kinder D Lasch J Willhardt I Sauer H Gaser C Phospholipase A2 activity is associated with structural brain changes in schizophrenia. Neuroimage 2010 52 1314 1327 10.1016/j.neuroimage.2010.05.009 20478385

[B6] Pearce JM Komoroski RA Mrak RE Phospholipid composition of postmortem schizophrenic brain by 31P NMR spectroscopy. Magn Reson Med 2009 61 28 34 10.1002/mrm.21820 19097198 PMC2610237

[B7] Horrobin DF Schizophrenia as a prostaglandin deficiency disease. Lancet 1977 1 936 937 67391 10.1016/s0140-6736(77)92228-0

[B8] Oresic M Hänninen VA Vidal-Puig A Lipidomics: a new window to biomedical frontiers. Trends Biotechnol 2008 26 647 652 10.1016/j.tibtech.2008.09.001 18951641

[B9] Cannon TD Kaprio J Lonnqvist J Huttunen M Koskenvuo M The genetic epidemiology of schizophrenia in a Finnish twin cohort. A population-based modeling study. Arch Gen Psychiatry 1998 55 67 74 10.1001/archpsyc.55.1.67 9435762

[B10] Spitzer RL Williams JB Gibbon M First MB The Structured Clinical Interview for DSM-III-R (SCID). I: History, rationale, and description. Arch Gen Psychiatry 1992 49 624 629 10.1001/archpsyc.1992.01820080032005 1637252

[B11] American Psychiatric Association Diagnostic and Statistical Manual for Mental Disorders 1994 4 Washington, DC: American Psychiatric Association

[B12] Cannon TD Hennah W van Erp TG Thompson PM Lönnqvist J Huttunen M Gasperoni T Tuulio-Henriksson A Pirkola T Toga AW Kaprio J Mazziotta J Peltonen L Association of DISC1/TRAX haplotypes with schizophrenia, reduced prefrontal gray matter, and impaired short- and long-term memory. Arch Gen Psychiatry 2005 62 1205 1213 10.1001/archpsyc.62.11.1205 16275808

[B13] Andreasen NC Negative symptoms in schizophrenia. Definition and reliability. Arch Gen Psychiatry 1982 39 784 788 10.1001/archpsyc.1982.04290070020005 7165477

[B14] Andreasen NC Olsen S Negative v positive schizophrenia. Definition and validation. Arch Gen Psychiatry 1982 39 789 794 10.1001/archpsyc.1982.04290070025006 7165478

[B15] Andreasen NC Carpenter WT Jr Kane JM Lasser RA Marder SR Weinberger DR Remission in schizophrenia: proposed criteria and rationale for consensus. Am J Psychiatry 2005 162 441 449 10.1176/appi.ajp.162.3.441 15741458

[B16] Cannon TD Huttunen MO Lönnqvist J Tuulio-Henriksson A Pirkola T Glahn D Finkelstein J Hietanen M Kaprio J Koskenvuo M The inheritance of neuropsychological dysfunction in twins discordant for schizophrenia. Am J Hum Genet 2000 67 369 382 10.1086/303006 10880296 PMC1287184

[B17] Surface Curve Protocol. http://www.loni.ucla.edu/~esowell/edevel/new_sulcvar.html

[B18] Medial Lines Protocol. http://www.loni.ucla.edu/~esowell/edevel/MedialLinesProtocol.htm

[B19] Pluskal T Castillo S Villar-Briones A Oresic M MZmine 2: Modular framework for processing, visualizing, and analyzing mass spectrometry-based molecular profile data. BMC Bioinformatics 2010 11 395 10.1186/1471-2105-11-395 20650010 PMC2918584

[B20] Nygren H Seppanen-Laakso T Castillo S Hyotylainen T Oresic M Liquid chromatography-mass spectrometry (LC-MS)-based lipidomics for studies of body fluids and tissues. Methods Mol Biol 2011 708 247 257 10.1007/978-1-61737-985-7_15 21207295

[B21] The R Project for Statistical Computing. http://www.r-project.org/

[B22] Kampstra P Beanplot: a boxplot alternative for visual comparison of distributions. J Stat Soft 2008 28 1 9

[B23] Fraley C Raftery AE Model-based methods of classification: Using the mclust software in chemometrics. J Stat Soft 2007 18 1 13

[B24] Zito T Wilbert N Wiskott L Berkes P Modular Toolkit for Data Processing (MDP): a Python data processing framework. Front Neuroinform 2008 2 8 19169361 10.3389/neuro.11.008.2008 PMC2628591

[B25] Castelo R Roverato A Reverse engineering molecular regulatory networks from microarray data with qp-graphs. J Comp Biol 2009 16 213 227 10.1089/cmb.2008.08TT 19178140

[B26] Brohee S Faust K Lima-Mendez G Vanderstocken G van Helden J Network Analysis Tools: from biological networks to clusters and pathways. Nat Protoc 2008 3 1616 1629 10.1038/nprot.2008.100 18802442

[B27] Orešič M Tang J Seppänen-Laakso T Mattila I Saarni SE Saarni SI Lönnqvist J Sysi-Aho M Hyötyläinen T Perälä J Suvisaari J Metabolome in schizophrenia and other psychotic disorders: a general population-based study. Genome Med 2011 3 e19 10.1186/gm233 PMC3092104 21429189

[B28] Suvisaari JM Saarni SI Perälä J Suvisaari JV Härkänen T Lönnqvist J Reunanen A Metabolic syndrome among persons with schizophrenia and other psychotic disorders in a general population survey. J Clin Psychiatry 2007 68 1045 1055 10.4088/JCP.v68n0711 17685741

[B29] Kirkpatrick B Miller BJ Garcia-Rizo C Fernandez-Egea E Bernardo M Is abnormal glucose tolerance in antipsychotic-naive patients with nonaffective psychosis confounded by poor health habits? Schizophr Bull 2012 38 280 284 10.1093/schbul/sbq058 20558532 PMC3283143

[B30] Guest PC Schwarz E Krishnamurthy D Harris LW Leweke FM Rothermundt M van Beveren NJ Spain M Barnes A Steiner J Rahmoune H Bahn S Altered levels of circulating insulin and other neuroendocrine hormones associated with the onset of schizophrenia. Psychoneuroendocrinology 2011 36 1092 1096 10.1016/j.psyneuen.2010.12.018 21251762

[B31] Guest PC Wang L Harris LW Burling K Levin Y Ernst A Wayland MT Umrania Y Herberth M Koethe D van Beveren JM Rothermundt M McAllister G Leweke FM Steiner J Bahn S Increased levels of circulating insulin-related peptides in first-onset, antipsychotic naive schizophrenia patients. Mol Psychiatry 2010 15 118 119 10.1038/mp.2009.81 20098438

[B32] Spelman LM Walsh PI Sharifi N Collins P Thakore JH Impaired glucose tolerance in first-episode drug-naive patients with schizophrenia. Diabet Med 2007 24 481 485 17381506 10.1111/j.1464-5491.2007.02092.x

[B33] Kotronen A Velagapudi VR Yetukuri L Westerbacka J Bergholm R Ekroos K Makkonen J Taskinen M-R Oresic M Yki-Järvinen H Saturated fatty acids containing triacylglycerols are better markers of insulin resistance than total serum triacylglycerol concentrations. Diabetologia 2009 52 684 690 10.1007/s00125-009-1282-2 19214471

[B34] Kaddurah-Daouk R McEvoy J Baillie RA Lee D Yao JK Doraiswamy PM Krishnan KRR Metabolomic mapping of atypical antipsychotic effects in schizophrenia. Mol Psychiatry 2007 12 934 945 10.1038/sj.mp.4002000 17440431

[B35] Kotronen A Yki-Jarvinen H Fatty liver: a novel component of the metabolic syndrome. Arterioscler Thromb Vasc Biol 2008 28 27 38 17690317 10.1161/ATVBAHA.107.147538

[B36] Pietiläinen KH Sysi-Aho M Rissanen A Seppänen-Laakso T Yki-Järvinen H Kaprio J Oresic M Acquired obesity is associated with changes in the serum lipidomic profile independent of genetic effects-a monozygotic twin study. PLoS ONE 2007 2 e218 10.1371/journal.pone.0000218 17299598 PMC1789242

[B37] Lagarde M Bernoud N Brossard N Lemaitre-Delaunay D Thies F Croset M Lecerf J Lysophosphatidylcholine as a preferred carrier form of docosahexaenoic acid to the brain. J Mol Neurosci 2001 16 201 204 discussion 215-221 10.1385/JMN:16:2-3:201 11478375

[B38] Amminger GP Schafer MR Papageorgiou K Klier CM Cotton SM Harrigan SM Mackinnon A McGorry PD Berger GE Long-chain {omega}-3 fatty acids for indicated prevention of psychotic disorders: a randomized, placebo-controlled trial. Arch Gen Psychiatry 2010 67 146 154 10.1001/archgenpsychiatry.2009.192 20124114

[B39] Oken RJ Schulzer M At issue: schizophrenia and rheumatoid arthritis: the negative association revisited. Schizophr Bull 1999 25 625 638 10667736 10.1093/oxfordjournals.schbul.a033407

[B40] Fuchs B Schiller J Wagner U Hantzschel H Arnold K The phosphatidylcholine/lysophosphatidylcholine ratio in human plasma is an indicator of the severity of rheumatoid arthritis: investigations by 31P NMR and MALDI-TOF MS. Clin Biochem 2005 38 925 933 10.1016/j.clinbiochem.2005.06.006 16043165

[B41] Buka SL Tsuang MT Torrey EF Klebanoff MA Bernstein D Yolken RH Maternal infections and subsequent psychosis among offspring. Arch Gen Psychiatry 2001 58 1032 1037 10.1001/archpsyc.58.11.1032 11695949

[B42] Torrey EF Yolken RH The schizophrenia-rheumatoid arthritis connection: infectious, immune, or both? Brain Behav Immun 2001 15 401 410 10.1006/brbi.2001.0649 11782106

[B43] Takatera A Takeuchi A Saiki K Morioka I Yokoyama N Matsuo M Blood lysophosphatidylcholine (LPC) levels and characteristic molecular species in neonates: prolonged low blood LPC levels in very low birth weight infants. Pediatr Res 2007 62 477 482 10.1203/PDR.0b013e31814625ca 17667851

[B44] Drobnik W Liebisch G Audebert FX Frohlich D Gluck T Vogel P Rothe G Schmitz G Plasma ceramide and lysophosphatidylcholine inversely correlate with mortality in sepsis patients. J Lipid Res 2003 44 754 761 10.1194/jlr.M200401-JLR200 12562829

[B45] Yan JJ Jung JS Lee JE Lee J Huh SO Kim HS Jung KC Cho JY Nam JS Suh HW Kim YH Song DK Therapeutic effects of lysophosphatidylcholine in experimental sepsis. Nat Med 2004 10 161 167 10.1038/nm989 14716308

[B46] Lusis AJ Atherosclerosis. Nature 2000 407 233 241 10.1038/35025203 11001066 PMC2826222

[B47] Yetukuri L Söderlund S Koivuniemi A Seppänen-Laakso T Niemelä PS Hyvönen M Taskinen MR Vattulainen I Jauhiainen M Oresic M Composition and lipid spatial distribution of HDL particles in subjects with low and high HDL-cholesterol. J Lipid Res 2010 51 2341 2351 10.1194/jlr.M006494 20431113 PMC2903811

[B48] Suvisaari JM Saarni SI Perala J Suvisaari JV Harkanen T Lonnqvist J Reunanen A Metabolic syndrome among persons with schizophrenia and other psychotic disorders in a general population survey. J Clin Psychiatry 2007 68 1045 1055 10.4088/JCP.v68n0711 17685741

[B49] Kubota M Miyata J Yoshida H Hirao K Fujiwara H Kawada R Fujimoto S Tanaka Y Sasamoto A Sawamoto N Fukuyama H Murai T Age-related cortical thinning in schizophrenia. Schizophr Res 2011 125 21 29 10.1016/j.schres.2010.10.004 21036016

[B50] Rund BR Melle I Friis S Johannessen JO Larsen TK Midboe LJ Opjordsmoen S Simonsen E Vaglum P McGlashan T The course of neurocognitive functioning in first-episode psychosis and its relation to premorbid adjustment, duration of untreated psychosis, and relapse. Schizophr Res 2007 91 132 140 10.1016/j.schres.2006.11.030 17258891

