# Supplementary material for: Phospholipids and insulin resistance in psychosis: a lipidomics study of twin pairs discordant for schizophrenia
Source: Genome Med. 2012 Jan 18;4(1):1. doi: 10.1186/gm300 (PMC3334549; doi:10.1186/gm300)
Supplement: Additional file 1 — Cognitive test scores. Table showing cognitive test scores with group comparisons. [file gm300-S1.PDF]

Cognitive test scores

Table showing cognitive test scores with group comparisons.

Variable name	Short notation	Controls		Co-twins		Patients		Controls *vs.* co-twins	Controls *vs.* patients	Co-twins *vs.* patients
		Mean	SD	Mean	SD	Mean	SD	Cohen's d	Cohen's d	Cohen's d
WMS-R Logical Memory Immediate	LMem-I	-0.2	(0.9)	-0.6	(0.7)	-1.1	(0.9)	0.45	0.95	0.59
WMS-R Logical Memory Delayed	LMem-D	0.0	(0.8)	-0.5	(0.8)	-0.9	(1.1)	0.61	1.06	0.47
WMS-R Visual Reproduction Immediate*	VRep-I	-0.2	(0.9)	-0.3	(0.9)	-1.4	(2.0)	0.09	0.84	0.72
WMS-R Visual Reproduction Delayed*	VRep-D	-0.8	(1.2)	-0.9	(1.3)	-2.3	(1.9)	0.12	0.98	0.80
WMS-R Digit Span Total	DSpan	-0.3	(0.7)	-0.8	(0.8)	-1.1	(0.8)	0.67	1.09	0.41
WMS-R Visual Span Total	VSpan	-0.3	(1.0)	-0.8	(0.9)	-1.1	(1.1)	0.56	0.82	0.29
WCST Categories Achieved*	WCST-CA	4.6	(1.7)	3.9	(2.7)	3.0	(3.0)	0.37	0.82	0.32
WCST Perseverative Errors*	WCST-PE	16.5	(11.5)	17.1	(8.7)	27.0	(27.1)	0.18	0.42	0.27
WAIS-R Vocabulary	Vocab	11.6	(2.3)	10.7	(2.3)	10.2	(3.1)	0.38	0.56	0.21
WAIS-R Similarities	Simil	10.4	(1.9)	10.3	(1.7)	9.5	(2.8)	0.09	0.44	0.35
WAIS-R Digit Symbol	DSym	13.7	(2.5)	11.7	(2.6)	9.1	(3.3)	0.81	1.67	0.89
WAIS-R Block Design	Blocks	11.4	(2.6)	10.1	(2.7)	9.1	(3.7)	0.51	0.78	0.32
CVLT Learning List A 1-5 Total	CVLT-A	49.9	(7.8)	49.8	(9.5)	40.0	(14.5)	0.02	0.96	0.82
CVLT Long Delay Free Recall	CVLT-LD	10.5	(2.6)	11.2	(2.8)	9.1	(4.2)	0.27	0.43	0.61
CVLT Recognition Hits*	CVLT-R	14.6	(1.1)	14.2	(1.6)	13.6	(2.5)	0.15	0.35	0.18
Trail Making Task A Response Time*	TMT-A	33.3	(11.1)	34.3	(16.2)	51.9	(36.1)	0.34	0.21	0.39
Trail Making Task B Response Time *	TMT-B	84.5	(33.0)	106.4	(59.2)	148.1	(106.0)	0.23	0.71	0.39
Finger Tapping Sum Left+Right	Tapping	119.9	(16.8)	111.7	(15.8)	101.8	(37.5)	0.51	0.79	0.38
COWAT Phonemic Fluency - Letters S+K	PhonFl	35.2	(11.1)	33.2	(9.4)	26.9	(10.9)	0.20	0.77	0.63

COWAT Semantic Fluency - Animals	SemFl	24.4	(7.2)	20.2	(4.3)	19.2	(6.4)	0.67	0.77	0.20
Stroop Color Word Response Time	Stroop	89.8	(14.2)	106.0	(20.9)	105.1	(14.8)	1.05	1.10	0.05

*Effect size calculated from scores transformed to approximately normal distributions. Transformed scores were used in all analyses.

Note: All effect sizes employ pooled standard variations.
