# Supplementary material for: Phospholipids and insulin resistance in psychosis: a lipidomics study of twin pairs discordant for schizophrenia
Source: Genome Med. 2012 Jan 18;4(1):1. doi: 10.1186/gm300 (PMC3334549; doi:10.1186/gm300)
Supplement: Additional file 3 — Selected independent components from MRI. Table showing selected independent components derived from MRI, shown for patients, their co-twins, patients and their co-twins combined (discordant twin pairs), and the controls. [file gm300-S3.PDF]

Selected independent components from MR imaging

Table showing selected independent components derived from MRI, shown for patients, their co-twins, and the controls.

Variable	Description	Controls	Co-twins	Patients	***P*** (F test)
IC2*	Right precentral areas	-9.5 (-13.2, -5.8)	-4.0 (-7.7, -0.3)	-3.0 (-7.5, 1.4)	0.15
IC3	Prefrontal areas	-10.0 (-14.7, -5.2)	-9.2 (-14.4, -4.0)	-5.5 (-10.0, -0.9)	0.46
IC5	Superior temporal gyri	10.2 (6.8, 13.7)	6.8 (1.8, 11.9)	6.6 (2.7, 10.5)	0.29
IC8	Frontal eye fields	-10.4 (-13.7, -7.1)	-16.4 (-18.8, -14.1)	-13.7 (-19.7, -7.7)	0.078
IC11	Frontal areas	-10.6 (-13.0, -8.2)	-6.9 (-11.7, -2.0)	-10.5 (-13.5, -7.4)	0.37
IC12	Prefrontal and superior temporal areas	0.7 (-2.8, 4.2)	4.2 (-0.4, 8.9)	3.0 (-1.8, 7.8)	0.52
IC13	Frontal areas	5.0 (2.2, 7.8)	8.9 (4.5, 13.4)	4.6 (1.7, 7.6)	0.29
IC15	Frontal areas	6.8 (3.8, 9.8)	10.7 (7.2, 14.2)	8.6 (4.8, 12.3)	0.35
IC17	Frontal and pre/postcentral areas	7.4 (4.4, 10.5)	8.2 (2.2, 14.1)	8.1 (5.2, 10.9)	0.95
IC18	Prefrontal areas	-0.2 (-3.0, 2.6)	-1.2 (-6.4, 4.1)	0.0 (-3.5, 3.4)	0.90
IC24	Left parietal areas	6.1 (3.6, 8.6)	2.7 (-0.6, 6.1)	3.0 (-1.2, 7.1)	0.30
IC25	Left sensorimotor and cingulate areas	-0.3 (-3.3, 2.7)	3.7 (0.3, 7.1)	-1.0 (-4.2, 2.2)	0.17
IC29	Prefrontal, lateral	7.0 (3.9, 10.1)	4.9 (0.9, 8.9)	8.7 (4.7, 12.7)	0.47

	temporal, and right cingulate areas				
IC30	Middle and inferior frontal areas	13.2 (10.6, 15.9)	15.9 (13.1, 18.7)	18.6 (14.6, 22.6)	0.11

*Data for this and other variables shown as mean (95% confidence interval), all values are multiplied by 1,000.
